# Animal Welfare and Policy Reforms for Korean Traditional Bull Fighting: Harmonizing Traditions with Animal Rights

**DOI:** 10.3390/ani15233440

**Published:** 2025-11-28

**Authors:** Gina S. Rhee, Rahyeon Ahn

**Affiliations:** 1 The Catholic College, The Catholic University of Korea, Bucheon 14662, Gyeonggi-do, Republic of Korea; 2 Department of Small Animal Surgery and Medicine, Ontario Veterinary College Health Sciences Centre, Guelph, ON N1G 2W1, Canada; rahyeonahn@gmail.com

**Keywords:** animal welfare, traditional practices, bullfighting, Korea, cultural heritage, policy reform, public subsidy, behavioral assessment, ethical transition

## Abstract

This study examines the welfare conditions, legal ambiguities, and economic inefficiencies inherent in Korea’s traditional bullfighting practices. The study analyzes field data collected during 2025 (February–June), covering 131 contests across six venues, collected by the Animal Liberation Wave (ALW); the results reveal pervasive welfare violations, as evidenced by high avoidance (41.2%) and injury (62.3%) rates among bulls, alongside notable physiological and behavioral stress markers. From a legal perspective, the Animal Protection Act of Korea displays a core inconsistency: it prohibits cruelty to animals yet exempts bullfighting on cultural grounds, thereby compromising legal coherence and undermining welfare standards. Public opinion surveys further demonstrate declining societal support, particularly among younger and urban populations. Comparative cases from Spain, Mexico, and the European Union illustrate alternatives and possible reforms that may preserve cultural identity while eliminating harm against animals. Accordingly, this study proposes a phased policy framework comprising immediate welfare oversight, gradual redirection of subsidies toward humane cultural programs, and legislative amendment to remove the exemption clause. Ultimately, this study contends that harmonizing Korea’s cultural heritage with international welfare norms is both ethically significant and legally necessary, offering a model for culturally sensitive reform in the global context.

## Simple Summary

The study explores the animal welfare problems and policy issues linked to Korea’s traditional bullfighting. Legally, Korea’s Animal Protection Act bans cruelty to animals but allows bullfighting as a “folk game.” This contradiction weakens the law’s consistency. Economically, the practice depends heavily on government subsidies and gambling revenue, offering little real profit or tourism benefit. Public opinion is turning against bullfighting, especially among young and urban citizens who view it as outdated and cruel. The authors recommend several reforms: strict welfare oversight, legal amendments to remove cultural exemptions, and redirecting public funds toward humane cultural festivals that honor Korea’s heritage without harming animals. The authors further argue that Korea can preserve its cultural identity while meeting international ethical standards, becoming a global example of reform that respects both tradition and animal welfare.

## 1. Introduction

### 1.1. Research Context and Significance

Animal welfare has emerged as a major policy concern across industrialized societies, reshaping longstanding associations between humans and nonhuman animals [1,2]. The growing attention paid to animal welfare globally reflects advances in animal behavior science and welfare assessment as well as broader moral and legal transformations that increasingly recognize animals as sentient beings entitled to protection. Therefore, the intersection between traditional cultural practices and contemporary animal welfare is a complex ethical challenge experienced by modern societies globally [1,2]. In this context, South Korea has experienced a remarkable transformation from a developing nation focused on economic growth to a prosperous democracy that is increasingly concerned with issues related to quality of life, environmental protection, and ethical considerations [3]. Over the past two decades, shifts in public consciousness regarding animal sentience, along with evolving legal frameworks and heightened moral expectations, have generated pressure to reconsider cultural practices that cause animal suffering. Thus, traditional practices that once escaped scrutiny under the banner of heritage are now subject to renewed ethical questions and policy debates.

In this regard, Korean traditional bull fighting occupies a particularly contested position. Korean bull fighting traces its origins to agricultural communities during the Joseon Dynasty (1392–1910), where cattle were considered symbols of wealth, social status, and community identity in addition to mere livestock [4,5]. Institutionalization began in the 1970s when Jinju, South Gyeongsang Province, hosted the first national-scale competition; furthermore, the introduction of local autonomy systems in the 1990s offered impetus as municipal and provincial governments began to adopt bull fighting as an economic and cultural tourism resource. Some individuals have celebrated it as a vibrant marker of local identity and cultural heritage, tracing its roots to agrarian rituals where such contests symbolized fertility, agricultural productivity, and communal solidarity [4,5]. Korean bull fighting differs fundamentally from Western bullfighting. In Korean bull fighting, two bulls are placed in an arena where they engage in head-to-head confrontations, pushing and clashing horns until one bull retreats, collapses, or is judged by officials to have lost dominance. There are no matadors, banderilleros, or ritualized killing as seen in Spanish bullfighting. Instead, trainers (called uju, meaning ‘bull owners’) use ropes (referred to as salkojul) attached to the bulls’ noses or horns to manipulate positioning and force continued engagement when animals attempt to disengage or flee [6]. Contests typically last between 5 and 30 min and are judged based on dominance displays, with victory awarded to the bull that forces its opponent to turn away or retreat. While Western bullfighting involves human–animal confrontation culminating in the bull’s death for ritual and spectacle, Korean bull fighting involves bull-to-bull combat. Bulls are not killed in the arena; however, severely injured or ‘retired’ bulls are often slaughtered for meat when their competitive careers end. Critics have identified it as a systemic form of cruelty that is incompatible with contemporary ethical and scientific understandings of animal well-being [7,8].

Modernization and globalization have further complicated the cultural significance of bull fighting. Bull fighting, which once served as a localized form of rural entertainment, has, over the past several decades, evolved into a commercialized, state-subsidized spectacle that relies heavily on gambling revenue and municipal funding [9,10]. These debates have strengthened Korean bull fighting as a cultural symbol tied to community pride while rendering it vulnerable to ethical, scientific, and legal challenges. Recent public opinion research demonstrates that these tensions are increasingly resolved in favor of reform: younger generations (ages 20–39) and urban populations show significantly higher opposition to bull fighting compared to older and rural demographics, with 62.1% of respondents expressing concern that the practice negatively influences children’s moral development [11]. This generational divide suggests that the cultural legitimacy of bull fighting is eroding as Korean society modernizes and adopts values more aligned with international animal welfare norms. Thus, Korean bull fighting stands at the crossroads of cultural preservation, public finance, and ethical reform, raising urgent questions about animal welfare, economic efficiency, and legal coherence. These dynamics illustrate why bull fighting functions as a critical case for examining how societies navigate the tension between cultural continuity and modern ethical standards.

### 1.2. Objectives and Scope

Drawing directly on the comprehensive investigative dataset published by ALW, this study undertakes a systematic re-examination and synthesis of the organization’s field findings with the explicit aim of informing policy discourse and proposing viable alternative frameworks.

First, drawing on the dataset generated through ALW’s systematic field investigations, the study presents a comprehensive documentation and quantification of welfare-related violations based on ALW’s rigorous recording of behavioral indicators of stress—including avoidance behaviors, defensive postures, and escape attempts—together with detailed classifications of the type, severity, and frequency of physical injuries and the full spectrum of handler-induced coercive practices observed across all 131 officially held contests nationwide.

Second, the study critically analyzes the legal inconsistencies embedded within the current regulatory regime, focusing on the tension between the Animal Protection Act’s overarching prohibition of animal cruelty and its categorical exemption for bullfighting as a “folk game,” and assesses the implications of this exemption for legal coherence and normative integrity in light of international legislative standards.

Lastly, building upon the empirical patterns revealed in the ALW dataset and the structural characteristics of the practice, the study advances evidence-based reform strategies by drawing on international precedent cases and formulating policy alternatives that seek to harmonize cultural heritage preservation with contemporary animal welfare principles, thereby providing a constructive foundation for legislative revision and institutional reform. Precise definitions and explanations of the methods used for on-site observation and recording are a must. Without these, interpretation of the presented results is compromised. This will also contribute to addressing the issues raised in Section 4.

### 1.3. Description of Korean Bullfighting Practice

Korean bullfighting involves staged confrontations between two bulls within an enclosed arena. Unlike Spanish-style bullfighting, Korean bullfighting is bull-versus-bull combat rather than human-versus-bull. Contests typically proceed as follows:

- Pre-contest: Bulls are transported to holding facilities 1–3 days before contests, restrained by nose rings (*kotture*) connected to ropes, and confined in narrow concrete pens averaging 6.44 m^2^ per animal.
- Arena entry: Handlers lead bulls into the arena using ropes attached to nose rings. Bulls are positioned facing each other at close range.
- Engagement: Handlers use ropes to pull bulls’ heads together, forcing frontal contact. If bulls attempt to disengage, handlers use various methods to sustain confrontation: shouting (observed in 89.3% of contests), prodding with poles/sticks (73.1%), and forceful rope manipulation (95.4%).
- Contest continuation: Contests continue until one bull retreats, refuses to re-engage, or is deemed defeated by judges. Confrontations involve head-to-head collisions, horn impacts, and pushing. Injuries commonly include horn lacerations, nasal bleeding, forehead wounds, and musculoskeletal damage.
- Outcome: Winners receive prize money; bulls may fight multiple times per season. Post-contest veterinary care typically consists of superficial wound cleaning and topical treatments oriented toward rapid return to competition rather than comprehensive recovery.
- Betting system: At Cheongdo permanent stadium, spectators purchase betting tickets and receive payouts based on contest outcomes, similar to horse race betting.

## 2. Literature Review

### 2.1. Welfare Science and Cattle

The literature consistently affirms cattle’s capacity to experience physical suffering and complex emotional and cognitive states. Across the fields of veterinary science, ethology, and animal behavior, studies have revealed that bovine welfare must extend far beyond mere avoidance of pain, encompassing the domains of fear, stress, and frustration [12,13]. Stress responses in cattle include physiological markers (e.g., elevated cortisol levels, increased heart rate, and altered respiration) and behavioral indicators (e.g., salivation, defensive posturing, and vocalization) [14,15]. Cortisol measurement represents the gold standard for stress assessment, with baseline levels of 0.5–2.0 ng/mL in unstressed cattle increasing by 5–10 times during acute stress episodes [16,17]. The stress responses documented in bull fighting contexts can be meaningfully compared to those observed in cattle confronting natural predators. Ethological studies of cattle under predation threat demonstrate that the primary behavioral response is flight rather than fight: when threatened by predators such as wolves or large carnivores, cattle preferentially flee to safety, with confrontation occurring only when escape routes are blocked [15,18]. Crucially, even when cattle do engage in defensive aggression against predators, such encounters are brief (typically lasting seconds to minutes) and terminate when the threat is neutralized or escape becomes possible [18]. In contrast, bull fighting contests artificially prolong confrontational stress by preventing flight: trainers use ropes to physically restrain bulls from retreating, and arena enclosures eliminate escape routes that would be available in natural settings. This forced prolongation of stress exposure represents a fundamental departure from natural cattle behavioral ecology, likely intensifying physiological stress responses beyond those experienced during predator encounters. The chronic nature of training regimens (bulls undergo repeated sparring sessions over months to years) further compounds this stress, creating conditions of repeated inescapable confrontation that have no natural analog in bovine evolution. These findings highlight that cattle are acutely sensitive to their environment and to social interactions that threaten their sense of security. Particularly relevant to Korean bull fighting is the literature on aggression and social hierarchies among cattle. Ethological studies report that while cattle establish dominance relationships within herds, these are generally established through ritualized, brief confrontations rather than sustained, violent conflicts [19]. Most cattle, when given the opportunity, will avoid rather than prolong confrontational situations, suggesting that extended fighting contests are inconsistent with their natural behavioral repertoire. Furthermore, forced encounters can result in chronic stress, impaired immune function, and increased vulnerability to injury [20]. Thus, the conditions imposed in bull fighting directly contradict the scientific understanding of bovine behavior and welfare, underscoring the systemic nature of the distress involved.

### 2.2. Cultural Continuity and Change

Anthropological research emphasizes that cultural practices are inherently adaptive, evolving in response to broader social, economic, and ethical transformations. Culture is continually renegotiated, with traditions persisting not due to the material practices themselves but because of the symbolic meanings and identities they embody [21,22]. This recognition provides a foundation for reimagining practices such as bull fighting in ways that preserve their communal significance while eliminating harmful elements.

There are several historical examples of societies that successfully transitioned from practices that inflicted suffering on animals or marginalized groups toward humane alternatives while maintaining cultural continuity. For example, Handler and Linnekin [23] demonstrated how traditions survive by adapting to new contexts, and DeMello [24] documented the transformation of ritual practices into symbolic or performative expressions that retain cultural resonance without perpetuating violence. Thus, in Korea, bull fighting can be considered a candidate for transformation into symbolic pageantry or festival-based alternatives. In such models, nonviolent performances, educational programs, or dramatized re-enactments could preserve the communal pride, local identity, and celebratory functions associated with the practice. This approach would align traditional customs with the broader principle that cultural resilience is expressed through innovation and re-articulation of meaning [25].

### 2.3. Legal Frameworks

The legal scholarship increasingly recognizes animals as beings with intrinsic moral and legal status. Theories of animal rights have highlighted that animals should not be treated as property or instruments for human use but as subjects of moral concern who deserve protection under law [26,27]. In the case of Korea, however, the Animal Protection Act reflects a profound contradiction. Article 8(2) of the Animal Protection Act of the Republic of Korea prohibits acts that inflict injury on animals for purposes such as gambling, advertisement, entertainment, or amusement. However, subparagraph 3 of the same paragraph explicitly exempts folk games prescribed by Ordinance of the Ministry of Agriculture, Food and Rural Affairs. Under the current Enforcement Decree of the Animal Protection Act and related ministry ordinances, the only practice that qualifies for this exemption is traditional Korean bullfighting 

Historically, dogfighting and cockfighting were also practiced as regional folk entertainment, but following the major amendment of the Animal Protection Act in 2018 (Act No. 16060) and the significant strengthening of penalties, both practices were completely banned. Consequently, among animal-versus-animal fighting practices, only bullfighting continues to enjoy legal protection through the Special Act on Traditional Bullfighting. and the aforementioned exemption clause in the Animal Protection Act. This selective retention of the bullfighting exemption, while dogfighting and cockfighting remain unconditionally prohibited, reveals an inconsistent application of cultural heritage justification.

Furthermore, this exemption undermines the spirit and the application of the law, suggesting that animal welfare protections remain conditional, and eroding coherence from the perspective of legal theory. Dworkin [28] argued that the legitimacy of law depends on its internal consistency: exemptions that allow cruelty for cultural purposes undermine that coherence and weaken public trust in the legal system. Comparative insights have further underscored this weakness. For example, the EU obliges its member to consider animal welfare in policymaking through Article 13 of the Treaty on the Functioning of the European Union (TFEU). Germany and Switzerland go even further, embedding animal dignity in their constitutions and thus enshrining welfare as a fundamental legal principle [29]. By contrast, Korea’s exemption for bull fighting as a folk game undermines domestic credibility and its international reputation, suggesting a reluctance to fully integrate animal welfare into the legal order.

### 2.4. Economic Perspectives

Cultural economics offers another perspective for evaluating practices, including bull fighting. Scholars have argued that public subsidies for cultural traditions should generate measurable community benefits, whether in the form of economic returns, social cohesion, or cultural education [30]. Evidence from Korea suggests that bull fighting fails to meet this criterion. The practice remains deeply dependent on municipal and national subsidies, with tourism revenues decreasing and gambling dominating the income stream. These economic patterns show that bull fighting operates at a net loss to society and crowds out opportunities for more sustainable and inclusive cultural investments.

Research has further highlighted that festivals and cultural events aligning with contemporary ethical standards frequently yield greater economic multipliers than controversial practices. Richards [31] demonstrated that cultural festivals rooted in heritage, food, and music can attract international visitors and produce substantial tourism spillovers. Cohen [32] and Andersson and Getz [33] similarly highlighted that ethical, family-friendly cultural events foster repeat visitation, improve community image, and promote long-term economic sustainability. In contrast, Korean bull fighting’s reliance on gambling undermines its cultural legitimacy while narrowing its economic benefits. This finding suggests that redirecting subsidies toward humane options could yield greater returns, both economically and socially, while aligning with global trends toward ethical cultural tourism.

### 2.5. Comparative Reforms

Originally developed by the UK Farm Animal Welfare Committee, the “Five Freedoms” framework has become the international standard for welfare assessment, encompassing freedom from hunger and thirst; freedom from discomfort; freedom from pain, injury, or disease; freedom to express normal behavior; and freedom from fear and distress [18,34]. However, recent advances in welfare science have highlighted positive welfare states rather than merely the absence of negative experiences, with animal welfare journals demonstrating the importance of affective states in comprehensive welfare assessment [35,36]. Comparative international cases have demonstrated that reform of animal-related traditions is feasible and often beneficial. Spain offers a complex example. In 2010, Catalonia prohibited bullfighting on animal welfare grounds; however, national courts later reinstated exemptions under claims of cultural identity [37]. This conflict illustrates the tension between cultural rights and welfare obligations while indicating that meaningful prohibitions are possible when public support aligns with political will. Mexico provides another instructive case. In Mexico City, the introduction of “bloodless bullfighting” retained ritual and symbolic elements while eliminating direct harm to animals [38]. Table 1 provides a comparative overview of these reform cases, including Catalonia, Mexico City, India’s Jallikattu regulation, and South Korea’s current status, demonstrating the range of policy approaches adopted across different cultural and legal contexts.

These cases highlight that reform is sustainable and compatible with cultural preservation. They reveal a spectrum of possible approaches, from outright bans to symbolic adaptations, each shaped by local context and public values.

In Korea, these precedents underscore the viability of developing festival-based or symbolic alternatives to traditional bull fighting. Such reforms allow for preserving communal pride and regional identity while aligning traditional practices with ethical imperatives and international expectations. By drawing on comparative experience, Korea can craft a reform pathway in a manner that balances cultural preservation with ending animal cruelty.

## 3. Methodology

### 3.1. Study Design and Data Sources

This study is grounded in field investigation data collected by Animal Liberation Wave (ALW) during February–June 2025. The investigation covered 131 bullfighting contests across 6 venues, 3 bull breeding farms in Cheongdo County, Cheongdo permanent bullfighting stadium, and other Folk festival contests in Uiryeong, Changnyeong, Changwon, and Daegu Dalseong districts in South Korea. For each contest, data included the date, venue, and contest number; the presence or absence of avoidance behaviors exhibited by the animals; the presence, absence, and severity of injuries sustained; the types and frequency of handler interventions; and the contest outcome (e.g., clear winner, mutual withdrawal, or other resolutions).

Photographic and video documentation provided visual corroboration across the events. This material captured clear evidence of injuries, stress-related behaviors, and prevailing environmental conditions at the venues, as well as documentation of handling practices during the contests and subsequent animal transport.

Environmental observations focused on the welfare conditions in holding facilities and event arenas. These included measurements of space allocation per animal, flooring type, restraint methods, and qualitative assessments of noise levels, crowding, and sanitation standards.

Economic and regulatory data were sourced from official records. This encompassed government subsidy figures for the Cheongdo bullfighting festival (obtained from Cheongdo County public records, 2020–2024, https://www.cheongdo.go.kr/budget/contents.do?mid=0318010000, accessed on 2 August 2025), attendance and revenue statistics from Cheongdo Public Corporation annual reports, and relevant legal documents and municipal ordinances governing the events.

### 3.2. Data Collection Methods

According to the report published by ALW, the field observation protocol encompassed full documentation of each contest, beginning with the arrival of the bulls and continuing through all stages of the event, including post-contest handling. Observers employed standardized field forms to record behavioral responses, visible injuries, handler interventions, and environmental conditions, while photographic and video materials were collected to ensure verification and consistency of the recorded data. The dataset represents a complete census of all 131 contests held across the six operational venues during the study period, thereby eliminating any concerns regarding selective sampling. All field investigators underwent formal training in bovine ethology and welfare assessment, which included systematic instruction in bovine stress indicators, injury classification frameworks, and standardized recording procedures. Additionally, observers consulted regularly with veterinary advisors and animal behavior specialists to enhance the accuracy and reliability of their assessments.

### 3.3. Operational Definitions of Key Variables

According to the report, avoidance behaviors were defined as instances in which bulls turned away from their opponents, attempted to leave the arena, refused re-engagement, or displayed persistent retreat, and these behaviors were recorded as present or absent for each contest. Injury assessments were categorized into three levels: superficial, moderate, and severe.

Superficial injuries, including minor horn abrasions and surface wounds; moderate injuries, such as visible lacerations or bleeding from the nasal passages or forehead; and severe injuries, encompassing limping, musculoskeletal damage, or collapse. Defensive postures were characterized by head-lowering with the body angled away, pawing at the ground, or adopting a lateral stance to avoid direct frontal contact. Escape reactions included attempts to exit the arena, turning away during active confrontation, or collapsing when overwhelmed. A fall was operationally defined as a loss of balance resulting in the bull lying down or being unable to maintain a standing position. Handler interventions—such as shouting, prodding with sticks or poles, and forceful manipulation of ropes—were systematically documented by frequency for each contest.

## 4. Results

### 4.1. Welfare Violations

Analysis of contest results revealed frequent and significant welfare violations. Avoidance behaviors, operationally defined as observable attempts to retreat from the opponent by moving backward, turning the body away from confrontation (presenting flank or hindquarters), lowering the head defensively while backing away, attempting to exit the arena, or refusing to re-engage when prompted by handlers pulling on ropes, were observed in 41.2% of contests. This percentage was calculated by dividing the number of contests with documented avoidance by the total number of observed contests. Bovine ethological literature demonstrates that cattle naturally avoid sustained aggressive confrontations; dominance hierarchies in natural settings are typically established through brief, ritualized displays rather than prolonged combat [19,20]. When given opportunity, cattle preferentially retreat from confrontational situations rather than prolong them. The high rate of avoidance observed in the study aligns with this ethological evidence, suggesting that extended fighting contests contradict natural bovine behavioral repertoires and that sustained aggression requires artificial inducement through handler intervention. Such behaviors challenge the claim that bull fights reflect natural dominance struggles, suggesting instead that the animals perceive these encounters as threatening and primarily seek to disengage. In addition to the pronounced avoidance and reluctance behaviors described above, physical injuries resulting from horn-to-horn and horn-to-body contact were commonly observed during and immediately after the contests [11]. Veterinary intervention, when present, was minimal, typically comprising topical treatments or superficial cleaning intended to facilitate rapid return to fighting rather than genuine recovery. The ALW report does not document whether analgesics (pain relievers) were administered to injured bulls.

The absence of documented analgesic protocols raises significant welfare concerns. If analgesics are not administered, bulls continue to experience pain during and after contests, prolonging suffering and potentially causing chronic pain that affects their quality of life during holding periods between contests; and if analgesics are administered without documentation, this may enable bulls to fight beyond their natural pain tolerance thresholds, masking injury severity and increasing risks of exacerbating musculoskeletal damage that could lead to chronic lameness or necessitate premature slaughter. Both scenarios represent welfare failures: untreated pain constitutes unnecessary suffering, while pain masking to prolong competitive use instrumentalizes animals’ bodies for human entertainment at the expense of their well-being. The lack of transparency regarding pain management protocols further undermines claims that bull fighting is conducted with adequate animal welfare oversight [11].

### 4.2. Welfare Assessment Using the Five Domains Framework

Animal welfare was evaluated using the Five Domains Model [36], which provides a structured approach encompassing five domains: (1) Nutrition, (2) Physical Environment, (3) Health, (4) Behavioral Interactions, and (5) Mental State.

Domain 1—Nutrition: Nutrition was not systematically assessed in the present study. Visual observation suggested that bulls were provided with adequate feed and water during holding periods.

Domain 2—Physical Environment: This domain was severely compromised. Bulls were confined in holding pens offering minimal space per animal, with concrete flooring and little or no bedding, restrictive tethering that prevented lying down or normal postural adjustments, and poor ventilation.

Domain 3—Health: Significant health impairments were evident. Bulls sustained multiple physical injuries during contests, most commonly horn-inflicted wounds, bruising, and musculoskeletal trauma. Post-contest medical treatment was limited and focused primarily on enabling the animal to return to competition quickly rather than ensuring full recovery.

Domain 4—Behavioral Interactions: Natural behavioral expression was severely restricted. Bulls were unable to engage in normal social, exploratory, or locomotor behaviors and were forcibly placed into aggressive confrontations that contradict their species-typical avoidance responses. Handler interventions (shouting, prodding, and rope pulling) were routinely used to compel continued fighting.

Domain 5—Mental State: Multiple indicators of negative affective states were observed, including excessive salivation, tachypnoea, defensive posturing, and escape attempts, pointing to experiences of fear, frustration, pain, and distress.

In summary, the Five Domains assessment reveals pervasive and severe welfare compromise across four of the five domains (physical environment, health, behavioral interactions, and mental state), with nutrition being the only domain without clear evidence of major concern in this context.

### 4.3. Stress Indicators

The contests were characterized by extensive behavioral indicators of stress. Salivation was noted in the observed bulls, frequently coupled with frothing at the mouth, a common marker of acute physiological strain. Excessive salivation and frothing at the mouth were frequently observed in the bulls. In livestock and animal welfare literature, excessive salivation and foam formation in cattle are primarily established as physiological indicators of heat stress [39,40]. Under heat stress conditions, cattle increase their respiratory rate for thermoregulation, during which panting is accompanied by excessive salivation and drooling [39]. Smith et al. reported that beef steers exposed to temperature fluctuations exhibited behavioral signs of extreme heat stress, including extended tongue protrusion, reduced appetite, excessive salivation, and increased respiratory rate [41]. Shephard comprehensively reviewed that elevations in rectal temperature, respiratory rate, drooling, sweating, and reduction in dry matter intake are the primary physiological indicators of heat stress in cattle [40]. Notably, Collier et al. explained that hypersalivation during panting represents a major pathway of sodium loss, with saliva being wasted through drooling and slobbering [39]. Rapid and labored breathing, recorded in the cases, further reinforced the conclusion of systemic stress responses [11]. Defensive posturing, including head-lowering, pawing at the ground, and lateral body positioning, were observed in the contests, suggesting heightened fear and attempts at self-protection [11]. Flight responses, including turning away from opponents, attempting to escape the arena, and outright collapse when overwhelmed, were documented in the cases [11]. Trainers consistently intervened to sustain engagement, shouting, prodding with sticks or poles, and forcefully manipulating ropes [11]. These interventions highlight the artificiality of sustained aggression, revealing that without coercion, several contests would have ended prematurely. The persistence of such practices illustrates that the apparent “willingness” of bulls to fight is in fact manufactured.

### 4.4. Economic Performance

The economic performance of bull fighting contests revealed an increasing dependence on state support and diminishing returns. Government subsidies increased from KRW 5.7 billion in 2020 to KRW 9.68 billion in 2024, an approximately 70% increase over 4 years [11]. Despite this investment, the net profit in 2024 was only KRW 59 million, generating a 0.61% ROI. Attendance figures declined by approximately 15% from 2022 to 2024, indicating declining public interest [11]. Moreover, the revenue structure was significantly skewed: gambling accounted for approximately 70% of the total income, whereas ticket sales, tourism, and local business spillovers contributed far less. This reliance on betting undermines claims that bull fighting primarily functions as cultural tourism. Instead, the data suggest that it operates as a subsidized gambling industry with minimal broader economic benefit, raising questions about the efficiency of public expenditure.

## 5. Discussion

### 5.1. Systemic Welfare Failures

The documented patterns of high-frequency avoidance behaviors, widespread physical injuries, and pervasive stress indicators reveal systemic welfare failures rather than isolated incidents, challenging fundamental claims about the cultural authenticity and animal welfare compatibility of bull fighting. Bulls’ behavioral responses—characterized predominantly by retreat attempts rather than sustained aggression—align with ethological expectations for a prey species confronting inescapable threat, not with voluntary participation in ritualized dominance contests. Trainers consistently intervened to sustain engagement, shouting, prodding with sticks or poles, and forcefully manipulating ropes [11]. These interventions highlight the artificiality of sustained aggression, revealing that without coercion, many contests would have ended prematurely due to bulls’ natural preference for conflict avoidance. The persistence of such coercive practices demonstrates that the apparent ‘willingness’ of bulls to fight is in fact manufactured through human intervention, fundamentally undermining claims that bull fighting represents a natural expression of bovine dominance behavior. The high prevalence of stress indicators (e.g., excessive salivation, rapid respiration, defensive posturing, and repeated attempts to escape) further undermine claims that these contests reflect natural bovine behavior. Instead, these outcomes align with established welfare science, demonstrating that cattle undergoing acute stress exhibit physiological and behavioral disruptions [13,14,15].

To compel bulls to continue engaging, trainers regularly resort to shouting, prodding, and rope manipulation, confirming that aggression does not spontaneously emerge but must be manufactured and thereby eroding any narrative of cultural authenticity. These findings also expose the artificiality of framing bull fighting as a naturalistic contest, considering that bovine ethology suggests avoidance, rather than sustained confrontation, as the typical behavioral response [19]. Repeated injuries, compounds this picture, establishing a predictable and preventable pattern of cruelty.

### 5.2. Legal Contradictions and Incoherence

From a legal standpoint, Korea’s approach to bull fighting is inconsistent. The Animal Protection Act prohibits acts that inflict unnecessary suffering; however, it exempts bull fighting from its ambit on the grounds of cultural heritage and folk tradition. This contradiction undermines the law’s normative authority. Theories of legal coherence highlight the need for consistent principles across domains [28]. By privileging cultural exemption, Korea erodes its legal system’s credibility and diminishes the scope of its animal welfare legislation.

International comparisons highlight Korea’s anomalous position. The EU recognized animal sentience in Article 13 of the TFEU, requiring welfare considerations in policymaking. This recognition was extended by Germany and Switzerland to constitutional law by embedding the dignity of animals as a guiding principle [29]. By contrast, Korea’s reliance on folk game exemptions aligns it with jurisdictions that prioritize tradition over animal welfare, contradicting its otherwise progressive stance on animal protection. This inconsistency risks reputational harm, especially as Korea seeks to assert leadership in global governance and ethical policymaking.

Proponents of bullfighting argue that the practice represents intangible cultural heritage dating to the Joseon Dynasty, when cattle symbolized wealth, status, and community identity in agricultural societies. They contend that bullfighting preserves rural traditions, promotes regional identity, and constitutes a legitimate expression of cultural diversity deserving protection.

However, cultural heritage arguments must be weighed against several considerations. First, anthropological scholarship demonstrates that cultural practices evolve adaptively in response to changing social values and ethical understandings [22,23]. Traditions persist through meaning and identity rather than unchanged material practices, suggesting that symbolic alternatives can preserve cultural significance while eliminating harm [24].

Second, cultural arguments do not override welfare obligations in other sectors. Animal use in agriculture, research, and entertainment is regulated by welfare standards; cultural heritage status does not exempt these domains from ethical scrutiny. The legal exemption for bullfighting creates an inconsistency wherein cattle in bullfighting receive less protection than cattle in other contexts.

### 5.3. Economic Inefficiency and Opportunity Costs

Government subsidies for bullfighting expanded from KRW 5.7 billion in 2020 to KRW 9.68 billion in 2024, representing a 69.75% increase over 4 years. Despite this substantial public investment totaling over KRW 35 billion across 5 years (2020–2024), net profits in 2024 were only KRW 59.2 million, generating a 0.61% return on investment (ROI) [11].

Revenue structure analysis reveals problematic dependence on gambling: betting (*ukkwon*) sales in 2024 totaled KRW 30.4 billion, but approximately 70% (KRW 21.3 billion) was returned as prize payouts, leaving minimal net revenue [11]. Attendance declined by approximately 15% from 2022 to 2024 (2024 attendance 391,514 visitors, averaging 3838 daily) [11]. Additionally, Cheongdo Public Corporation pays over KRW 1.7 billion annually in facility usage fees to the private contractor (Korea Bull Fighting Association Co.), which uses these payments to service a KRW 20 billion loan used to construct the stadium. The corporation has also guaranteed this debt, exposing taxpayers to potential liabilities of hundreds of billions of won [11].

Continued subsidies were associated with substantial opportunity costs. The public funds directed toward bull fighting could support humane cultural events, agricultural heritage festivals, or sustainable tourism initiatives that align more closely with contemporary ethical standards and international market trends [31,32]. Such alternatives could yield higher returns, attract diverse audiences, and establish Korea as a nation committed to innovation and humane values.

#### Tourism Market Analysis and Reputational Risks

The economic justification for bull fighting often invokes tourism revenue; however, this claim requires critical examination of target market demographics and value alignments. International tourism to South Korea is heavily weighted toward visitors from Western nations (Europe, North America, Australia), China, and Southeast Asia, with cultural tourism (K-pop, traditional palaces, cuisine) representing the fastest-growing segment. Research on tourist attitudes toward controversial attractions demonstrates that ethical controversies can create reputational spillover effects that extend far beyond the specific site.

Studies of controversial tourism practices reveal patterns of reputational damage applicable to South Korea’s situation. Research on slum tourism demonstrates that controversial attractions presented in commercial tourism contexts can negatively affect tourists’ moral perceptions and create broader ethical concerns about the destination [42,43]. Crapolicchio et al. found that when tourism activities involve viewing vulnerable populations or ethically questionable practices, tourists’ evaluations of those practices—and their perceptions of the host community—are significantly influenced by how the activity is framed and commercialized [42]. Similarly, Shang et al. documented that tourists apply moral judgments when evaluating destinations where tourism involves controversial practices, with such judgments potentially affecting broader destination perceptions [43].

This pattern is further illuminated by research on dark tourism and controversial attractions more broadly. Gui and Zhong’s systematic review of slum tourism research reveals that when destinations commercialize controversial or ethically problematic activities, they risk creating negative associations that extend beyond the specific attraction to affect the destination’s overall image [44]. Assylkhanova et al. note in their critical review of dark tourism that sites associated with suffering, exploitation, or ethical controversies face ongoing challenges in managing visitor perceptions and destination reputation, particularly as global ethical standards evolve [45].

For South Korea, which has invested heavily in cultivating an international image as a technologically advanced, culturally sophisticated democracy, the association with animal fighting presents reputational risks. International condemnation of bull fighting could undermine Korea’s broader ‘soft power’ and cultural diplomacy efforts, particularly as the country seeks to position itself as a leader in ethical governance and sustainable development. The lesson from slum tourism and dark tourism research is clear: controversial attractions marketed for commercial gain can create ‘moral taint’ effects that discourage precisely the high-value tourists (educated, affluent, ethically conscious travelers) that cultural tourism strategies aim to attract.

Furthermore, even if bull fighting attracts some domestic and regional tourists, the demographic profile leans heavily toward older male gamblers rather than families, international visitors, or repeat cultural tourists who generate broader economic multipliers through accommodation, dining, and secondary attractions. The reliance on gambling revenue confirms that bull fighting functions primarily as a betting venue rather than a cultural tourism attraction, with minimal spillover benefits to local restaurants, hotels, or heritage sites.

### 5.4. Public Opinion

Public opinion surveys have confirmed increasing public disapproval of bull fighting in its current form. According to ALW [11], 70.2% of respondents opposed the continuation of gambling systems tied to the contests, reflecting widespread concern about the social consequences of state-supported betting. Furthermore, 62.1% of the respondents expressed concern about the potential negative moral impact of bull fighting on children, citing risks of desensitization to violence and normalizing cruelty [11]. Women, urban residents, and younger generations—groups that historically shape long-term social trends—exhibited the strongest opposition. Meanwhile, rural respondents, who have conventionally reported higher support for bull fighting, presented ambivalent attitudes: only a slight majority endorsed continuation, suggesting that the practice no longer enjoys unequivocal legitimacy even within rural communities. Collectively, these findings indicate a shifting moral landscape wherein bull fighting is increasingly perceived as inconsistent with contemporary values of animal welfare, child protection, and responsible cultural policy.

### 5.5. Social and Criminological Dimensions

Justifying the continuation of bull fighting from an ethical perspective is challenging. From a deontological perspective, the practice violates the imperative to not instrumentalize sentient beings merely for human entertainment [27]. Utilitarian analyses emphasize the disproportionate suffering inflicted on animals relative to the marginal and declining benefits for human participants [46]. Meanwhile, the capability theory underscores the deprivation of animals’ opportunities according to their species-specific capacities [47]. Collectively, these frameworks deem bull fighting as ethically indefensible.

Survey data reveal widespread concern that exposing children to organized violence against animals harms their moral development. Criminological research on illegal animal fighting provides relevant context, though important distinctions must be acknowledged. Burchfield analyzed animal crime patterns in Chicago, finding that when animal crimes co-occurred with other offenses, they were most frequently associated with drug or weapons violations, with animal crime offenders [48]. Walliss reviewed the social scientific literature on illegal dogfighting in the UK and USA, documenting how dogfighting operates as an underground criminal enterprise involving networks of participants engaged in training, breeding, and organizing fights, often connected to illegal gambling operations [49]. Siegel and van Uhm examined illegal dogfighting in the Netherlands, documenting connections to organized crime networks, particularly illegal gambling and money laundering; the authors note that “dog fights are often linked to organized crime, especially illegal gambling and money laundering,” creating economic incentives that sustain the practice despite legal prohibition [50]. Young conducted ethnographic research on cockfighting in rural Hawaii, examining how participants develop legal consciousness—their understanding of and relationship to law enforcement—in contexts where cockfighting is illegal but culturally embedded, illuminating how animal fighting can persist in legal gray zones where cultural tradition and legal prohibition intersect [51].

However, crucial differences limit the direct applicability of this research to Cheongdo bull fighting. First, the cited studies examine illegal activities operating in criminal contexts, whereas Cheongdo bull fighting operates under legal exemption with government support—a fundamental difference affecting participant demographics, social organization, and connections to other criminal enterprises. Second, none of these studies directly examined bull fighting or established causal connections between animal fighting and community violence, youth delinquency, or family violence; the research documents correlations in illegal contexts but cannot be extrapolated to make causal claims about legal animal fighting’s community impacts. Third, the studies examine Western contexts where animal fighting lacks cultural legitimacy and legal protection, differing from South Korea where bull fighting is defended as cultural tradition.

Despite these limitations, the criminological literature raises legitimate concerns applicable to South Korea’s context. The integration of legal gambling into bull fighting (70% of revenue) creates risks of gambling addiction, which is well-established as psychologically harmful and associated with family breakdown and financial distress. When gambling is structurally linked to animal suffering, it may create psychological associations between cruelty and reward that normalize both behaviors, particularly concerning for children who observe adults celebrating and betting on animal contests. Moreover, government subsidies and official sanction (through legal exemptions) send signals that violence against animals is culturally acceptable when framed as tradition or entertainment, potentially contributing to broader normalization of violence.

While the criminological research on illegal animal fighting cannot establish direct causal links to Cheongdo’s context, it highlights risks warranting ongoing monitoring: the potential for normalizing violence, the documented harms of gambling addiction, and the ethical implications of government sanction for practices causing severe animal suffering.

### 5.6. Policy Recommendations

The findings demonstrate that the current model of Korean bull fighting is unsustainable in terms of welfare, legality, and economics. Therefore, a comprehensive set of policy recommendations is needed to guide reform. The first priority is establishing immediate interventions that address the most egregious animal welfare violations. Independent veterinary oversight must be established at all bull fighting venues, with veterinarians empowered to halt contests in cases of injury or extreme distress. This measure would mitigate immediate suffering while aligning Korea with international welfare standards that emphasize professional monitoring as a baseline requirement [52]. 

This legal contradiction exemplifies what Han identifies as paradigmatic anomaly in jurisprudence [53]. Drawing on Kuhnian philosophy of science, the author argues that legal systems operate under dominant opinions that function as paradigms, efficiently resolving routine cases until anomalies accumulate and precipitate crisis. The bullfighting exemption constitutes such an anomaly: it cannot be reconciled with the Animal Protection Act’s foundational principle prohibiting cruelty, yet persists through cultural heritage justifications that increasingly conflict with contemporary ethical consciousness.

Furthermore, to mitigate social harms associated with exposing children to animal violence, restrictions on audience participation should be implemented. Research on moral development has highlighted that recurrent exposure to cruelty erodes empathy and normalizes aggression [54,55]. Prohibiting attendance for children under a defined age threshold, alongside parental consent for adolescents, could protect vulnerable groups while signaling a cultural shift away from the normalization of violence.

Beyond immediate reforms, transitional strategies are crucial for facilitating cultural continuity while eliminating detrimental practices. Public subsidies, which currently sustain bull fighting, should be progressively redirected toward alternative cultural programs that celebrate agricultural heritage without perpetuating cruelty. Such cultural events can include exhibitions of traditional cattle breeding, farming demonstrations, festivals of local food culture, and regional music and dance performances. Richards [31] and Cohen [32] demonstrated that festivals rooted in authentic cultural identity can attract diverse audiences, yield economic multipliers, and foster community pride. By reimagining bull fighting festivals as celebrations of agrarian heritage, Korea can preserve its regional identity while responding to and aligning with ethical imperatives. This process should be participatory and involve farmers, local governments, and cultural organizations in designing new forms of expression. The cultural resilience theory underscores that traditions endure not by rigid preservation but through adaptive innovation [22]. During this transition, a phased reallocation of subsidies would ensure economic stability while gradually reducing dependence on gambling revenues.

Legal reform is indispensable for ensuring coherence and sustainability. The exemption of bull fighting in the Animal Protection Act constitutes a significant contradiction that undermines the law’s credibility and weakens the state’s commitment to protecting animal welfare [28]. Eliminating or severely restricting this exemption would restore consistency and align Korea with jurisdictions that treat cultural heritage as subject to welfare standards rather than as a justification for cruelty. Comparative models in the EU, where proportionality tests balance cultural rights with welfare obligations, provide a valuable framework [29]. Such a strategy would enable Korean courts to evaluate claims of cultural significance while ensuring that the harms inflicted on animals are not dismissed as inconsequential. Over the long term, Korea should consider embedding animal dignity within constitutional law, similar to Germany and Switzerland, thereby securing a principled foundation for welfare policy that cannot be easily undermined by legislative exemptions.

Finally, the reform process should be viewed in a global context. By adopting humane alternatives and strengthening its legal framework, Korea has an opportunity to position itself as a leader in reconciling cultural heritage with modern welfare standards. Collaborations with the United Nations Educational, Scientific and Cultural Organization; EU institutions; and international NGOs could enhance the legitimacy of reforms, attract positive international attention, and establish Korea as a model for culturally sensitive welfare policy. Comparative cases from Spain and Mexico indicate that cultural practices associated with animal harm can be transformed or replaced without eroding identity or economic vitality [37]. By following these examples, Korea can preserve regional pride while upholding ethical obligations and contribute to the global evolution of cultural policy.

Overall, these recommendations form a comprehensive reform agenda that balances immediacy with long-term vision. Welfare oversight and child protection ensure speedy responses to ongoing harms; transitional strategies offer pathways for cultural adaptation and economic stability; legal reform ensures coherence and credibility; and international engagement elevates Korea’s role in shaping global standards. By pursuing these measures in tandem, Korea can transform a disputed and increasingly indefensible practice into an opportunity for cultural innovation, ethical leadership, and international recognition.

## 6. Conclusions

Korean bull fighting epitomizes the tension between cultural heritage and modern animal welfare standards. Based on data obtained from ALW, the results indicate widespread welfare violations, legal inconsistencies, economic inefficiencies, and social concerns. Bull fighting contests inflict systematic suffering, evidenced by avoidance behaviors, high injury rates, and visible stress. Economically, subsidies generate insignificant returns while public opinion demonstrates growing opposition.

International comparisons reveal that reforms are possible and can be beneficial. By transitioning toward humane cultural alternatives, Korea can preserve its symbolic traditions while aligning with ethical and legal standards. Comprehensive reforms—through welfare oversight, legal amendment, and cultural innovation—provides a path toward harmonizing tradition with compassion. In doing so, Korea can emerge as a leader in culturally sensitive animal welfare policy, setting an example for societies across the world that are grappling with achieving balance between heritage and humane treatment.

## Figures and Tables

**Table 1:** International Comparative Cases of Traditional Animal Practice Reforms: Status and Approaches.

Country /Region	Practice	Year of Major Reform	Current Status	Key Reform Approach	Cultural Continuity Maintained?
Catalonia (Spain)	Spanish-style bullfighting	2010 (Ban) 2016 (Partially Reinstated)	Banned in Catalonia and Canary Islands; Permitted elsewhere in Spain	Regional Parliamentary Ban	Partial (Bloodless or Symbolic Events in some Areas)
Mexico City (Mexico)	Bullfighting	2022 (Indefinite Suspension)	Suspended in Mexico City; Permitted in other States	Regional Parliamentary Ban	Yes (Symbolic Reenactments Allowed)
India (Tamil Nadu)	Jallikattu (Bull taming/ Vaulting)	2017 (Regulated after Supreme Court Ruling)	Strictly Regulated with Welfare Ordinances	Supreme Court Guidelines + State-level Animal Welfare Rules	Yes
South Korea	Bull- vs. Bull- fighting	No Major Reform	Fully permitted under “Folk Game” exemption	Cultural Exception Clause in Animal Protection Act	Yes (Unrestricted)

## Data Availability

The raw data supporting the conclusions of this article will be made available by the authors on request.
